# Large Anal Polyp Disguised as Rectal Prolapse

**DOI:** 10.7759/cureus.65193

**Published:** 2024-07-23

**Authors:** Anwar A Khan, Luis F Cervera, Sammy Shihadeh, Daniel Glotzer

**Affiliations:** 1 Clinical Sciences, Florida State University College of Medicine, Tallahassee, USA; 2 Surgery, Cleveland Clinic Indian River Hospital, Vero Beach, USA

**Keywords:** transanal excision of polyp, proctosigmoidectomy, rectal polyp, rectal prolapse, anal polyp

## Abstract

Rectal prolapse is a relatively rare condition where the rectal mucosa protrudes out of the anal canal. The diagnosis is made through a physical exam and clinical evaluation, and surgical treatment options can vary. Anal polyps masquerading as rectal prolapse have rarely been described in the literature. A 79-year-old man presented with a four-year history of a bulging, protruding mass from his anus that is exacerbated with defecation and bowel movements. He was initially diagnosed with rectal prolapse and had a proctosigmoidectomy performed robotically. Shortly after the procedure, his symptoms recurred, and he was referred to a different surgeon for reevaluation. A large, prolapsed polyp was visible on the physical exam. A colonoscopy and an anoscopy were performed. The CT abdomen/pelvis revealed a mass within the rectum, and the biopsy showed an adenomatous polyp with high-grade dysplasia. The patient underwent a transanal excision of the rectal polyp, with symptoms permanently resolving. For an accurate diagnosis, it is crucial to conduct a comprehensive assessment of the patient's history, a physical exam, and an unusual clinical course of rectal prolapse. The rarity of large, prolapsed polyps, along with their similar presentation to that of other anorectal conditions, may have contributed to this patient's diagnosis of rectal prolapse and the subsequent proctosigmoidectomy in place of a transanal excision of a polyp. The palpation of a stalk on a physical exam should raise suspicion of a polyp, and further workup, such as a colonoscopy and/or anoscopy, should be conducted to confirm the diagnosis.

## Introduction

Rectal prolapse is a relatively rare condition where the rectal mucosa protrudes out of the anal canal [1]. Although the condition mainly affects older women and is associated with dysfunction and defects in the pelvic floor musculature, older men can also be affected [2]. Patients present with a mass that prolapses through the anus, commonly through an increase in intra-abdominal pressure (Valsalva, bowel movements, etc.), that either reduces spontaneously or may require manual reduction. Patients can also report tenesmus, fecal incontinence, and bleeding, among other symptoms [1]. Laboratory values will be unremarkable but may show iron deficiency anemia. Rectal prolapse is a clinical diagnosis based on the patient's symptoms and physical examination findings; radiological evaluation by means of defecography may be required [3]. The severity and extent of surgical treatment options vary and depend on presenting symptoms, available resources, and physician and patient preference.

Rectal adenomas and polyps are relatively common conditions, with more than 40,000 individuals in the U.S. diagnosed with rectal cancer every year [4]. Adenomas are considered pre-malignant lesions, with an associated 8% risk of malignancy over the next five years [5]. Adenomatous polyps can appear anywhere along the rectum and present with signs similar to rectal prolapse, such as constipation, tenesmus, and bleeding. Polyps rarely reach a size that allows them to protrude into the anal canal. Large anal polyps masquerading as rectal prolapse and the association of rectal cancers with rectal polyp prolapse have rarely been described in the literature [6,7].

## Case presentation

A 79-year-old man with a history of prostatectomy secondary to prostate cancer, radiation therapy to the pelvis, and urinary incontinence presented with a four-year history of a bulging, protruding mass from the anus that is exacerbated by defecation and bowel movements. He reported that he manually reduced the mass after defecating and would occasionally notice blood in his stool. He was initially diagnosed with rectal prolapse and had a proctosigmoidectomy performed robotically. Shortly after the procedure, his symptoms recurred, and he was referred to a different surgeon for reevaluation.

**Figure 1:**
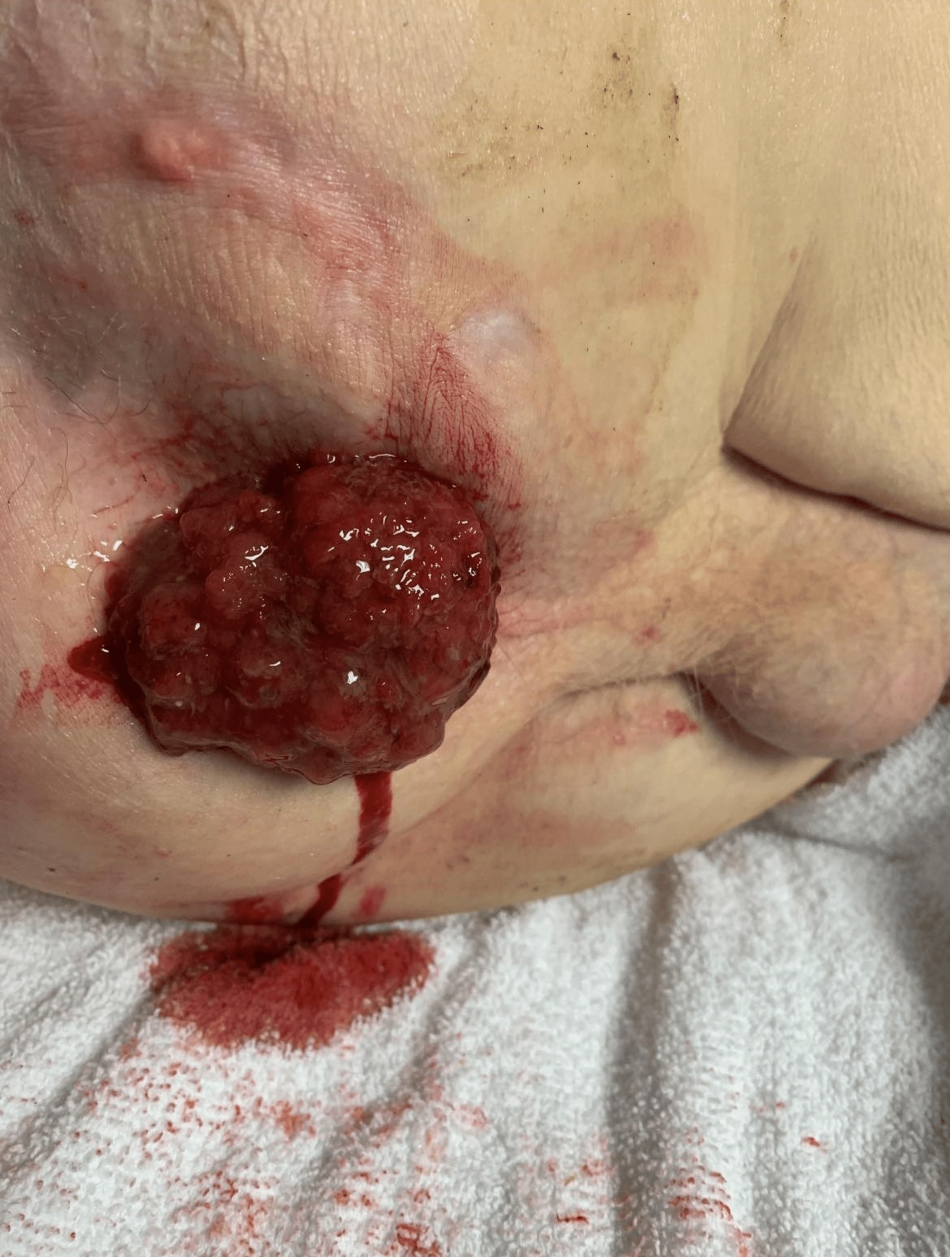
The patient in lateral decubitus position with a prolapsed rectal polyp

All vital signs and laboratory values were within the reference range. A large, prolapsed polyp was visible on the physical exam (Figure 1). A colonoscopy and an anoscopy were performed. A CT scan of the abdomen and pelvis revealed a 4.1 cm × 4.3 cm mass within the rectum, and a biopsy revealed a tubulovillous adenomatous polyp with high-grade dysplasia in which invasion could not be ruled out. Subsequently, the patient underwent a transanal excision of the rectal polyp, with symptoms permanently resolving. Although this patient presented with symptoms typical of rectal prolapse, a mass could be palpated within the rectum, raising suspicion for an alternative diagnosis.

## Discussion

Colon polyps are common within the elderly population, with 50% of those over the age of 70 having been affected by the condition [8]. Due to their silent clinical nature, the majority of rectal polyps are found during routine colonoscopy. Some rectal polyps may be found after the patient reports symptoms of bleeding, tenesmus, and, in severe cases, prolapse of the polyp. Malignant features of a rectal polyp include large size (>2 cm), friability, ulceration, and induration [4]. Rectal polyps, particularly those in the lower third of the rectum, have a high tendency to metastasize to lymph nodes and require radical surgical treatment [9]. Colonoscopy is the gold standard when evaluating rectal polyps and is sometimes supplemented with CT imaging [8].

The management of rectal polyps depends on multiple factors, including size, location, and histopathological features. Traditionally, smaller polyps located anywhere along the anal canal and rectum were managed through removal via colonoscopy, while larger polyps located in the lower rectum were managed surgically through transanal excision using anal retractors and diathermy [10]. Larger polyps located in the upper or middle thirds of the rectum were managed more aggressively using transabdominal resection with possible anastomoses due to their decreased visibility and inaccessibility using transanal approaches. Transanal endoscopic approaches are also utilized when possible as less invasive means in the management of such polyps [10].

The differential diagnoses for rectal prolapse include prolapsed rectal mucosa, internal hemorrhoids, intussusception, solitary rectal ulcer, and anal and rectal polyps [1]. A thorough history and physical exam and an unusual clinical course may help narrow the diagnoses. A confirmatory biopsy is required to differentiate adenomatous polyps from other similar-appearing lesions and to determine potentially malignant histopathological features. An anal polyp can rarely grow large enough to prolapse through the anal canal [4]. This unfamiliarity with large, prolapsed polyps, along with their similar presentation to that of other anorectal conditions, and the lack of a credible, conventional, shared, and approved clinical procedure (algorithm) may have contributed to this patient's diagnosis of rectal prolapse and the subsequent proctosigmoidectomy in place of a transanal excision of a polyp.

## Conclusions

The differential diagnosis for rectal prolapse should include a prolapsed anal and/or rectal polyp, among others. As anal polyps may be mistaken for rectal prolapse, a full clinical picture of the patient’s symptoms, history, and physical exam findings are paramount. The palpation of a stalk or polyp on a physical exam should raise suspicion of a polyp, and further workup, such as a colonoscopy and/or anoscopy, should be conducted to confirm the diagnosis. A biopsy and resection of the polyp should be performed to determine the histology and risk of malignancy and recurrence. The initial misdiagnosis in this patient may have occurred due to cognitive factors such as premature closure, as rectal prolapse is a much more common condition than anal polyps. This case presentation also demonstrates that when a patient's clinical course continues to worsen after the initial intervention, alternative diagnoses should be considered. It is crucial to be cautious of diagnostic pitfalls such as latent errors and anchoring biases to avoid premature diagnoses and unnecessary treatment.

## References

[1] Bordeianou L, Hicks CW, Kaiser AM, Alavi K, Sudan R, Wise PE (2014). Rectal prolapse: an overview of clinical features, diagnosis, and patient-specific management strategies. J Gastrointest Surg.

[2] Schey R, Cromwell J, Rao SS (2012). Medical and surgical management of pelvic floor disorders affecting defecation. Am J Gastroenterol.

[3] Dvorkin LS, Gladman MA, Epstein J, Scott SM, Williams NS, Lunniss PJ (2005). Rectal intussusception in symptomatic patients is different from that in asymptomatic volunteers. Br J Surg.

[4] Cowan ML, Silviera ML (2016). Management of rectal polyps. Clin Colon Rectal Surg.

[5] O'Brien MJ, Winawer SJ, Zauber AG (1990). The national polyp study. Patient and polyp characteristics associated with high-grade dysplasia in colorectal adenomas. Gastroenterology.

[6] Cano-Contreras AD, Meixueiro-Daza A, Grube-Pagola P, Remes-Troche JM (2016). Giant rectal polyp prolapse in an adult patient with the Peutz-Jeghers syndrome. BMJ Case Rep.

[7] Erikoğlu M, Tavli S, Tekin S (2004). A rare case of rectal prolapse associated with rectal adenocarcinoma: case report. Turk J Gastroenterol.

[8] Munteanu I, Mihaela M, Popescu S, Slavu IM, Oprescu Macovei A, Cochior D (2023). Giant villous adenoma of the rectum with prolapse: case report. Cureus.

[9] Nascimbeni R, Burgart LJ, Nivatvongs S, Larson DR (2002). Risk of lymph node metastasis in T1 carcinoma of the colon and rectum. Dis Colon Rectum.

[10] Heidary B, Phang TP, Raval MJ, Brown CJ (2014). Transanal endoscopic microsurgery: a review. Can J Surg.

